# The Emergency Medicine Resident Retreat: Creating and Sustaining a Transformative and Reflective Experience

**DOI:** 10.7759/cureus.27601

**Published:** 2022-08-02

**Authors:** Daniel J Egan, Chen He, Quinn Leslie, Mark A Clark, Resa E Lewiss

**Affiliations:** 1 Emergency Medicine, Harvard Medical School, Boston, USA; 2 Emergency Medicine, Icahn School of Medicine at Mount Sinai, New York City, USA; 3 Emergency Medicine, Schuyler Hospital, Montour Falls, USA; 4 Emergency Medicine, Thomas Jefferson University, Philadelphia, USA

**Keywords:** residency curriculum, residency innovation, residency program, residency wellness, resident retreat, emergency medicine resident

## Abstract

Introduction

Burnout rates for emergency medicine residents are high. One intervention and initiative to enhance wellness and address burnout is the resident retreat. Retreats have multiple formats and are often designed with an emphasis on social events. This longitudinal retreat curriculum for a three-year residency training program was designed emphasizing rest, a step away from what is familiar, and reflection.

Methods

Individual resident retreats were designed for each year of postgraduate training. The agenda for each is organized and intentional. Activities focused on personal well-being, self-reflection, team building, professional development, and physical activities are coupled with topics unique to class year roles and responsibilities. Retreats are held away from the hospital establishing a separation from the workplace.

Results

The retreat program has been sustainable for almost decades with trainees evaluating it highly. Faculty and residents enthusiastically participate in the program and consider it a fundamental part of the residency; 93.75% of residents surveyed strongly agreed that the retreats benefit their training while 94.2% strongly agreed that retreats increased their enthusiasm for training.

Conclusions

An emergency medicine resident retreat program focusing on unique elements for each post-graduate year is achievable and sustainable in an emergency medicine residency program. Over time, the retreat has become an integral part of the residency experience with positive experiences for both faculty and trainees.

## Introduction

The Accreditation Council for Graduate Medical Education (ACGME) requires residencies to incorporate well-being into the clinical work environment as well as scheduling, and accessibility to mental health resources [1]. Additionally, in response to three resident suicides in NYC in 2014, the ACGME Council of Review Committee Residents, a multidisciplinary group of residents and fellows from 29 specialties, made recommendations for the promotion of wellness and mental health awareness. These included “promoting a more supportive culture in training programs...including team building and resident retreats” [2]. 

Few reports exist on the successful implementation of a structured resident retreat program. In pediatrics, a retreat program at the University of Washington has evolved over 20 years to include curriculum components such as stress management, professional fulfillment attainment, and the management of feelings of incompetence during training [3]. Using a multidisciplinary approach with the Department of Humanities, Penn State has highlighted topics, which may act as triggers for burnout including communicating bad news, dealing with the dying patient, mistakes, “stress and distress” of residency, and the changing role of the physician in society [4]. 

Although up to 91% of program directors in emergency medicine report using retreats as a wellness initiative [5], only one emergency medicine retreat has been described in the literature. This focuses on the use of games for team building [6]. Others, including this model, have been presented as didactics at the Council of Residency Directors in Emergency Medicine Academic Assembly [7,8]. In this report, we describe an innovative strategy for an emergency medicine resident retreat in a post-graduate year (PGY) 1-3 training program in New York City. What makes this program design unique is the class-based approach and an intentional step away from what is familiar, allowing insight, rest, reflection, and an evolved way of viewing the current work environment. Many emergency medicine retreats focus on social rather than transformative experiences. While the social motivations of games, picnics, or parties build camaraderie, a successful retreat returns residents to the workplace with increased focus, motivation, and inspiration. Studies suggest that emergency medicine trainees experience burnout during training at rates from 62%-76% [9-12]. This program is designed to enhance wellness, inspire a re-connection with values and work as an emergency physician, boost morale and community, and provide practical tools for stress reduction and strategies for success in training. 

This work was originally presented at the 2015 Council of Residency Directors in Emergency Medicine Academic Assembly on April 14, 2015.

## Materials and methods

This work received exemption from the IRB at the Icahn School of Medicine at Mount Sinai.

To execute a longitudinal program with multiple retreats over the course of the academic year, it is critical to address multiple elements in the developmental phase: messaging, faculty involvement, agenda, location, finances, retreat timing, resident and faculty expectations. Program leadership must emphasize consistent messaging that retreats are not purely social and aim to achieve the objectives outlined above. Engaged and enthusiastic faculty serving as team members and facilitators is critical. Each retreat’s agenda incorporates some elements consistently: personal check-ins (inquiries on each person's well-being and life outside of the hospital), self-reflection questions (goals, achievements, vision), physical activities (games, hikes), professional development activities targeted to the postgraduate year (leadership activities, teaching, and supervisory skills), wellness topics (burnout discussions, mindfulness), and community building (Tables 1-4). Retreats are evaluated through anonymous surveys distributed each year as part of the internal annual program evaluation.

**Table 1:** Post-Graduate Year (PGY) 1 Retreat Agenda

PGY1 Retreat Agenda (Winter)	
8:00 AM	Homemade breakfast and personal check-in. Discussion on intern year so far.
9:30 AM	“What I wish I had been told as an intern” led by faculty moderators.
10:30 AM	Group hike outdoors
12:00 PM	Lunch with journal entry sharing
1:00 PM	Writing exercise: Setting personal and career goals
1:30 PM	Quiet time and self-reflection questions
2:30 PM	Strategies for success in the emergency department discussion: Difficult patients Being rushed and overwhelmed Emotionally charged cases Making mistakes Difficult consultants Dealing with your senior Asking for and getting helpful feedback Asking for and getting teaching
3:30 PM	Team building exercise
4:30 PM	Future directions and goals discussion: Taking ownership of your education Authenticity, integrity in professional life Cultivating professionalism Balance and healthy living
5:45 PM	Free time and dinner
6:30 PM	Depart

**Table 2:** Post-Graduate Year (PGY) 2 Overnight Retreat Agenda

PGY 2 Two-Day Retreat (Fall)	
8:00 AM	Arrival and room check-in
8:30 AM	Breakfast
9:30 AM	Introduction and personal check-in
10:30 AM	Team-building (Outdoor blind maze, asking for help)
11:30 AM	Discussion session: Life in the Emergency Department: The good, the bad, and the ugly
12:30 PM	Lunch
1:30 PM	Hike
4:30 PM	Free time
5:00 PM	Care packages
6:00 PM	Dinner Pair-share someone else’s story
7:00 PM	Film and discussion
9:00 PM	Open Mic/Talent Show
DAY TWO	
8:00 AM	Breakfast
9:00 AM	Wellness, burnout and strategies for success
10:00 AM	Mindfulness, restorative yoga
11:30 AM	Team feedback
12:30 PM	Lunch
1:15 PM	Free time
2:15 PM	Team building outdoor activity
3:15 PM	Quiet time and self-reflection questions
4:30 PM	Wrap-up
6:00 PM	Dinner
7:00 PM	Depart

**Table 3:** Rising Senior Resident Retreat Agenda

PGY 2 Rising Senior Retreat (Spring)	
8:00 AM	Homemade breakfast and personal check-in.
9:00 AM	Hike with breakout session mid-hike at lake: Successes as senior resident Challenges and practical solutions
12:00 PM	Lunch
1:00 PM	Quiet time and self-reflection questions
2:30 PM	Rising chiefs session
3:00 PM	Burnout, balance and resilience in emergency medicine
4:00 PM	Wrap-up
4:30 PM	Depart

**Table 4:** Graduating Senior Resident Retreat Agenda

PGY 3 Graduating Senior Retreat (Spring)	
9:00 AM	Homemade breakfast and personal check-in.
10:00 AM	Hike
11:00 AM	Pearls and pitfalls of life after residency
12:00 PM	Lunch and swimming
1:30 PM	Quiet time and goal clarification writing exercise
2:30 PM	Burnout, resilience, balance and growth in emergency medicine
3:30 PM	Free time
4:00 PM	Wrap up
4:30 PM	Depart

Based on years of collective leadership experience and reflection on the specific experiences of each post-graduate year, the program leadership designs the agendas to emphasize stressful situations and contributors to burnout unique to that year. The faculty wellness champion and program leadership reassess the agenda each year for appropriateness and relevance based on the current training environment. For messaging consistent with clarity, health, and wellness, we design the one-day retreats as alcohol-free. While we allow alcohol on the overnight retreat, consumption is de-emphasized and neither funded nor provided by the faculty or residency program.

The program requires resources (financial for food and/or facility rental, shift coverage, schedule adjustments) and departmental leadership support, without which it will not succeed. The location should minimize distractions, have outdoor space for activities, and serve as a peaceful respite, especially for urban residencies. Residents must leave the work setting to create a mental separation from the hospital. We choose faculty homes for single-day retreats, and a retreat center for the one overnight retreat in the PGY2 year. This longer and more comprehensive retreat is strategically placed in the middle of the three-year program. Doing so infuses energy and renewed dedication to training at a time when most residents begin to run out of steam; the novelty of internship is over and the completion of training seems far away and daunting.

We hold class-specific retreats throughout the year, always including the weekly protected conference time: PGY-1 in the winter to address seasonal challenges and burnout associated with that time of year, PGY-2 in the autumn to mark the midway mark for their residency, and again in the spring as a transition to senior year, and PGY3 in the spring preparing for graduation and the transition to academic or community practice. The retreat team (faculty wellness champion/retreat director, residency leadership, and resident director of wellness) reviews the agenda and identifies facilitators for each session. Residents are protected from clinical duties beginning overnight before the retreat until the morning after the retreat by other residents in the program. The tradition of retreats and benefit to each resident for their own relief of clinical duties allows for the sustainability of this model. The post-retreat protection from clinical shifts following completion of the agenda allows for protected time for socialization and class bonding. To get the most out of the experience, participants are asked to prepare and begin to enter the retreat mindset ahead of the event. The residents journal ten minutes after each shift for two weeks prior to the retreat. We offer limited guidance on the process and assure residents that they will not be required to share their journal entries. We provide a standardized burnout inventory for self-assessment prior to the retreat used by the residents for self-reflection prior to the event. 

## Results

Our first retreat was in 2005. Five years of annual program evaluation data using Likert scale assessments (2012-2013 and 2015-2017) has demonstrated the achievement of our objectives. Over this five-year period, 205 surveys were administered (45 residents each year) and 115 returned (56.1%); 93.75% of residents strongly agreed that the retreats benefitted their training, while 93.50% strongly agreed that retreats increased their enthusiasm for training and served as a useful intervention for goal setting. And 94.25% of trainees strongly agreed that the retreat program aided them in their current resident roles and helped them prepare for future responsibilities and careers; 94.75% strongly agreed that the retreat experience improved unity and mutual respect amongst peers. Finally, 91.75% of residents strongly agreed that the program enhanced their awareness of the importance of compassion and humanism in medicine.

Anecdotally, both resident and faculty participants have reported that the retreats allowed them to refresh and refocus. Many faculty members attend repeatedly, feeling that they get as much out of the program as they give to it. Graduates who have remained on the program’s faculty are often the first to volunteer as facilitators. The retreats have a cumulative effect moving from guarded discussions in the PGY-1 year to greater and deeper sharing of challenges, goals, and experiences in the PGY-2 and PGY-3 years commensurate with the development of a culture of support, safety, and camaraderie.

## Discussion

The overarching need to offer protected time and space for trainees to step away from daily responsibilities, recommit to core values, and gain new insight and inspiration is critical in graduate medical education. In a recent survey, emergency medicine has shifted to become the specialty with the highest degree of burnout [13]. Many healthcare leaders attribute such a high rate to the working conditions and risks of the COVID-19 pandemic [14-16], which has undoubtedly also impacted the experience and wellness of trainees. This sustainable retreat program has demonstrated success with resident satisfaction and is the first report in the literature for emergency medicine. A similarly organized radiology program using team building, guided reflection, and design thinking demonstrated similar outcomes [17]. As noted, our experience has shown that several components of the program truly enhance success: a retreat site that is separate from the training program’s clinical site, protection from clinical duties before and after, an emphasis on nutritious and health-conscious meals, and the creation of an environment where residents can truly step away and reflect upon their core values as physicians. Our success is complemented by dedicated faculty members with strong interests in wellness, the willingness to facilitate emotionally charged and sometimes difficult discussions, as well as enhance the ability to listen to and guide vulnerable participants. We coach our session leaders to facilitate but not dominate the conversation, to listen deeply, and to draw as many residents into the meaningful discussion as possible. Importantly, once on retreat, the residents must have psychological safety, assurance of confidentiality, and absence of retaliation by leadership for topics shared.

These undertakings require monetary and explicit support from the sponsoring department. Additionally, other trainees must value the program in order to accept schedule modifications to accommodate colleagues in other classes to attend their sessions. From a participant standpoint, trust in the program’s mission and process are crucial. The more skeptical of the retreat program and/or the less vulnerable and open the participants are during group discussions and guided reflection activities, the less successful the overall impact of the program will be. While the activities vary, a universal theme year to year is an emphasis on returning to the goals and values that initially attracted the residents to medicine, integrated with their current experiences in training. We see this process as an antidote for the burnout caused by a loss of meaning in work that occurs during training for many residents. The goal is to remind residents why they chose this arduous path and to provide avenues for them to reclaim those goals and values.

The retreat program is constantly evolving. We use feedback and experience from each retreat to improve subsequent ones. We trial new experiences including the involvement of a mediation expert, and the use of mental health practitioners for focused discussions on the trauma that emergency physicians experience. These thoughtful retreats are critical in establishing a meaningful wellness program and have been widely successful in our program. Our goals for next steps involve coaching to help residents incorporate the skills they gain on retreats into their daily lives and to encourage regular access to mental health care to facilitate more lasting wellness.

## Conclusions

Using a standardized curriculum across years of training, an emergency medicine retreat program is both sustainable and well-received. Each year of training presents different challenges for residents, which may contribute to decreased job satisfaction and increased rates of burnout. A protected social experience for residents can positively impact training, address challenges of professional identity, build community, and foster skills development for trainee well-being in emergency medicine.
